# Supplementary material for: PKA drives an increase in AMPA receptor unitary conductance during LTP in the hippocampus
Source: Nat Commun. 2021 Jan 18;12:413. doi: 10.1038/s41467-020-20523-3 (PMC7814032; doi:10.1038/s41467-020-20523-3)
Supplement: Supplementary file 1 — Supplementary Information [file 41467_2020_20523_MOESM1_ESM.pdf]

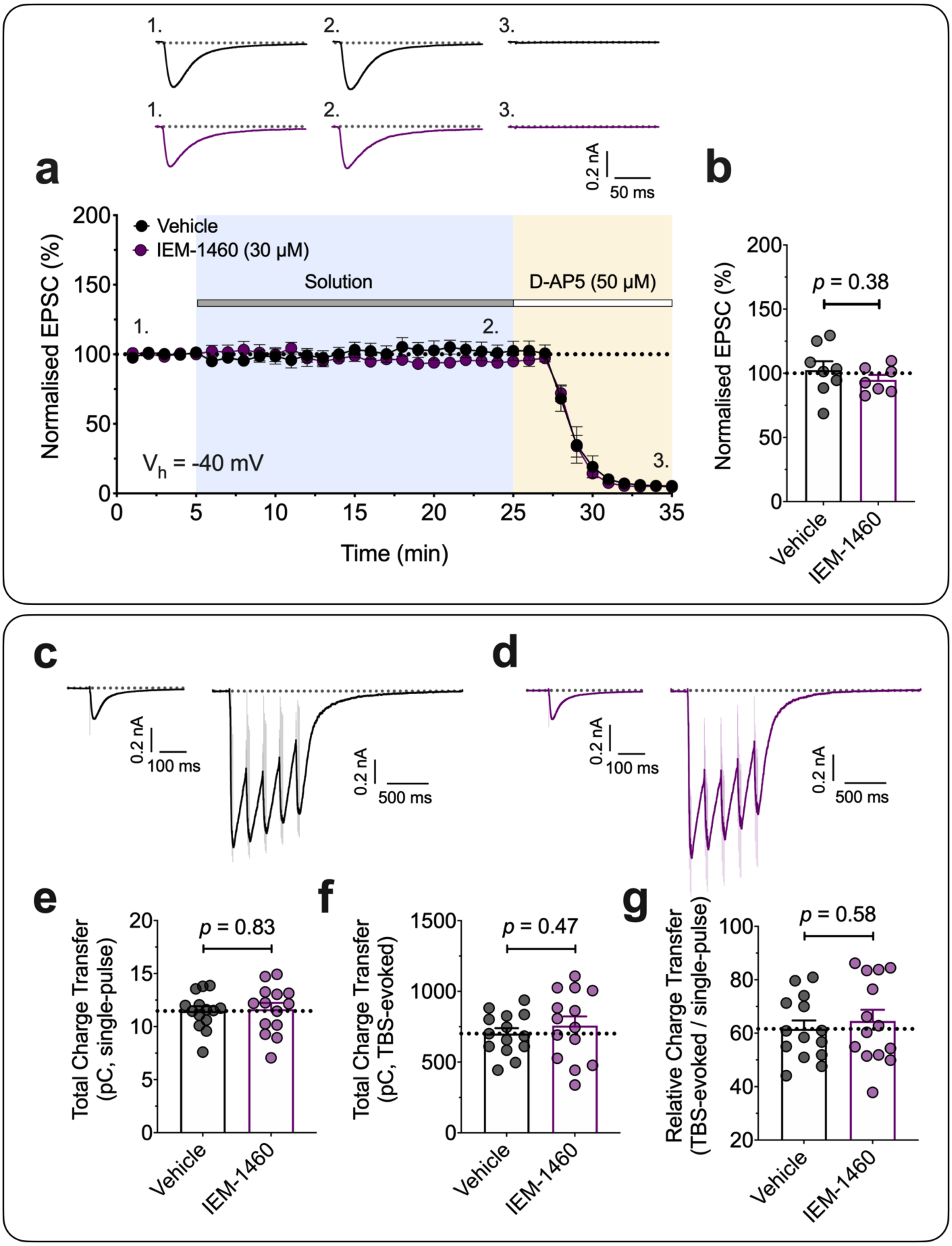

a
b
c
d
e
f
g
1.
2.
3.
0.2 nA
50 ms
Vehicle
IEM-1460 (30 μM)
Solution
D-AP5 (50 μM)
Normalised EPSC (%)
V_h = -40 mV
Time (min)
p = 0.38
100 ms
500 ms
Total Charge Transfer (pC, single-pulse)
p = 0.83
Total Charge Transfer (pC, TBS-evoked)
p = 0.47
Relative Charge Transfer (TBS-evoked / single-pulse)
p = 0.58

Supplementary Figure 1. Lack of effect for IEM on NMDAR-mediated currents. a, IEM-1460 (30 μM) was applied to slices while recording synaptic currents (inset) in the presence of picrotoxin (50 μM) and NBQX (10 μM) at a holding potential of -40 mV (mean ± SEM, n = 7 neurons from 6 animals for IEM and 8 neurons from 6 animals for vehicle control). Subsequent application of D-AP5 (50_μM) confirmed that the EPSCs were mediated by NMDARs. **b,** No significant difference was found after 20 min of vehicle control vs. IEM-1460 application (t_{13} = 0.9158, *p* = 0.3764, two-sided unpaired Student's *t*-test). **c-d,** Representative traces for currents evoked by single-pulse and TBS (i.e. 25 stimuli) for vehicle (**c**) and IEM-1460 (**d**) treated slices. Thin lines, original traces; thick lines, low-pass filtered at 200 Hz. **e-g,** Quantification for total charge transfer during single-pulse evoked currents (t_{26} = 0.2191, *p* = 0.8283, two-sided unpaired Student's *t*-test) (**e**), TBS-evoked currents (t_{26} = 0.7341, *p* = 0.4694, two-sided unpaired Student's *t*-test) (**f**), and the ratio of these two measurements (t_{26} = 0.5637, *p* = 0.5778, two-sided unpaired Student's *t*-test) (**g**) for vehicle (n = 14 neurons from 8 animals) and IEM-1460 (n = 14 neurons from 8 animals) treated groups. Data are presented as mean ± SEM. Source data are provided as a Source Data file.

Supplementary Table 1. Summary of EPSC properties for the various experimental protocols and conditions.

Protocol	γ (pS)		EPSC (%)	τ_{rise} (ms)		τ_{decay} (ms)		N
Compressed TBS	5.09 ± 0.32	5.34 ± 0.37	212 ± 11 ***	1.23 ± 0.06	1.20 ± 0.07	7.17 ± 0.19	7.07 ± 0.23	22/15
Spaced TBS	6.91 ± 0.44	8.40 ± 0.44 **	177 ± 9 ***	1.16 ± 0.06	1.15 ± 0.05	7.09 ± 0.19	6.75 ± 0.20 **	23/17
wTBS with rolipram	4.86 ± 0.43	8.02 ± 0.58 ***	234 ± 14 ***	1.22 ± 0.06	1.16 ± 0.05	7.19 ± 0.20	6.68 ± 0.15 ***	21/15
wTBS with PKA Cα	5.15 ± 0.51	7.79 ± 0.80 ***	276 ± 19 ***	1.02 ± 0.06	1.02 ± 0.04	6.75 ± 0.22	6.41 ± 0.23 *	17/13
wTBS with PKA Cα + IEM	4.29 ± 0.51	4.32 ± 0.54	202 ± 16 ***	1.00 ± 0.07	0.99 ± 0.07	6.73 ± 0.25	6.70 ± 0.26	16/13
HI-CaMKII	4.57 ± .053	4.57 ± 0.52	112 ± 7	1.01 ± 0.06	1.01 ± 0.07	6.99 ± 0.34	6.96 ± 0.25	14/11
CaMKII	4.71 ± 0.57	4.32 ± .046	169 ± 15 ***	0.95 ± 0.05	0.93 ± 0.04	6.82 ± 0.30	6.75 ± 0.29	15/12
CaMKII + PKA Cα	4.64 ± 0.39	6.48 ± 0.42 ***	178 ± 10 ***	1.05 ± 0.07	1.03 ± 0.08	7.06 ± 0.26	6.69 ± 0.19 *	18/15
CaMKII + PKA Cα + IEM	4.26 ± 0.33	4.13 ± 0.40	128 ± 9 *	1.01 ± 0.04	1.00 ± 0.04	6.91 ± 0.20	6.87 ± 0.22	20/15
HI-CaMKII + PKA Cα	4.24 ± 0.35	4.22 ± 0.40	112 ± 9	1.01 ± 0.03	0.97 ± 0.03	7.07 ± 0.27	7.05 ± 0.26	16/14
CaMKII + IEM	4.07 ± 0.39	4.04 ± 0.66	165 ± 17 **	0.97 ± 0.03	0.98 ± 0.03	6.75 ± 0.22	6.78 ± 0.34	8/8

*$p < 0.05$, **$p < 0.01$, ***$p < 0.001$ for baseline vs. LTP in two-sided paired Student's *t*-test.
