# Supplementary material for: PKA drives an increase in AMPA receptor unitary conductance during LTP in the hippocampus
Source: Nat Commun. 2021 Jan 18;12:413. doi: 10.1038/s41467-020-20523-3 (PMC7814032; doi:10.1038/s41467-020-20523-3)
Supplement: Supplementary file 3 — Reporting Summary [file 41467_2020_20523_MOESM3_ESM.pdf]

nature research

Corresponding author(s): Graham Collingridge

Last updated by author(s): Nov 17, 2020

Reporting Summary

Nature Research wishes to improve the reproducibility of the work that we publish. This form provides structure for consistency and transparency in reporting. For further information on Nature Research policies, see our Editorial Policies and the Editorial Policy Checklist.

Statistics

For all statistical analyses, confirm that the following items are present in the figure legend, table legend, main text, or Methods section.

n/a	Confirmed	
☐	☒	The exact sample size (*n*) for each experimental group/condition, given as a discrete number and unit of measurement
☐	☒	A statement on whether measurements were taken from distinct samples or whether the same sample was measured repeatedly
☐	☒	The statistical test(s) used AND whether they are one- or two-sided<br>*Only common tests should be described solely by name; describe more complex techniques in the Methods section.*
☐	☒	A description of all covariates tested
☐	☒	A description of any assumptions or corrections, such as tests of normality and adjustment for multiple comparisons
☐	☒	A full description of the statistical parameters including central tendency (e.g. means) or other basic estimates (e.g. regression coefficient) AND variation (e.g. standard deviation) or associated estimates of uncertainty (e.g. confidence intervals)
☐	☒	For null hypothesis testing, the test statistic (e.g. *F*, *t*, *r*) with confidence intervals, effect sizes, degrees of freedom and *P* value noted<br>*Give P values as exact values whenever suitable.*
☒	☐	For Bayesian analysis, information on the choice of priors and Markov chain Monte Carlo settings
☒	☐	For hierarchical and complex designs, identification of the appropriate level for tests and full reporting of outcomes
☒	☐	Estimates of effect sizes (e.g. Cohen's *d*, Pearson's *r*), indicating how they were calculated

Our web collection on statistics for biologists contains articles on many of the points above.

Software and code

Policy information about availability of computer code

Data collection	All recording data collected with WinLTP v2.3. Confocal images with Leica SP8 LAS X.
Data analysis	Data analyzed with WinLTP v2.3, Clampfit v10.1, Mini Analysis v6.0, WinWCP v5.1 and Graphpad Prism 8

For manuscripts utilizing custom algorithms or software that are central to the research but not yet described in published literature, software must be made available to editors and reviewers. We strongly encourage code deposition in a community repository (e.g. GitHub). See the Nature Research guidelines for submitting code & software for further information.

Data

Policy information about availability of data

All manuscripts must include a data availability statement. This statement should provide the following information, where applicable:
- Accession codes, unique identifiers, or web links for publicly available datasets
- A list of figures that have associated raw data
- A description of any restrictions on data availability

All data reported in this study are available from G.L.C. upon reasonable request. Source data are provided with this paper.

Field-specific reporting

Please select the one below that is the best fit for your research. If you are not sure, read the appropriate sections before making your selection.

☒ Life sciences ☐ Behavioural & social sciences ☐ Ecological, evolutionary & environmental sciences

For a reference copy of the document with all sections, see nature.com/documents/nr-reporting-summary-flat.pdf

Life sciences study design

All studies must disclose on these points even when the disclosure is negative.

Sample size	The number of experiments were based on previous publications in our labs as well as from other labs working on this topic of synaptic plasticity (e.g. Benke et al., Nature, 1998; Zhou et al., Nature Neuroscience, 2018).
Data exclusions	Whole-cell recordings were only accepted for analysis if access resistance varied by <15%. This was based on previous studies on this topic of synaptic plasticity and was necessary for an accurate measurement of AMPAR conductance (e.g. Benke et al., The Journal of Physiology, 2001). Exclusion criteria were pre-established.
Replication	The number of replication is noted in the figure legends, and all the reported data were reliably reproduced.
Randomization	Test and control groups were randomly assigned and interleaved at the start of experiment.
Blinding	Experiments were performed with knowledge of the group allocations. Blinding was not relevant to this study as experiments were required to control LTP induction protocols. Group allocations were blind during data analysis.

Reporting for specific materials, systems and methods

We require information from authors about some types of materials, experimental systems and methods used in many studies. Here, indicate whether each material, system or method listed is relevant to your study. If you are not sure if a list item applies to your research, read the appropriate section before selecting a response.

Materials & experimental systems

n/a	Involved in the study	
☒	☐	Antibodies
☐	☒	Eukaryotic cell lines
☒	☐	Palaeontology and archaeology
☐	☒	Animals and other organisms
☒	☐	Human research participants
☒	☐	Clinical data
☒	☐	Dual use research of concern

Methods

n/a	Involved in the study	
☒	☐	ChIP-seq
☒	☐	Flow cytometry
☒	☐	MRI-based neuroimaging

Eukaryotic cell lines

Policy information about cell lines

Cell line source(s)	Lenti-X 293T cell line was obtained from Takara Bio.
Authentication	Lenti-X 293T cell line was not authenticated after being obtained from Takara Bio.
Mycoplasma contamination	The cell line was not tested for mycoplasma contamination.
Commonly misidentified lines (See ICLAC register)	No commonly misidentified cell line was used.

Animals and other organisms

Policy information about studies involving animals; ARRIVE guidelines recommended for reporting animal research

Laboratory animals	Male C57BL/6 mice (2-3 months of age) only for CRISPR experiments and male Sprague-Dawley rats (1-3 months of age) for other experiments.
Wild animals	No wild animals used.

Field-collected samples	No field-collected samples used.
Ethics oversight	UK Animals (Scientific Procedures) Act of 1986, and Institutional Animal Care and Use Committees in Seoul National University.

Note that full information on the approval of the study protocol must also be provided in the manuscript.
